# Supplementary material for: Functional heterogeneity and plasticity in naïve CD8 T cells drive superior effector and memory responses
Source: bioRxiv. 2025 Oct 17:2025.10.16.682909. Preprint. [Version 1] doi: 10.1101/2025.10.16.682909 (PMC12632747; doi:10.1101/2025.10.16.682909)
Supplement: 1 [file NIHPP2025.10.16.682909V1-supplement-1.pdf]

bioRxiv preprint doi: https://doi.org/10.1101/2025.10.16.682909; this version posted October 17, 2025. The copyright holder for this preprint (which was not certified by peer review) is the author/funder, who has granted bioRxiv a license to display the preprint in perpetuity. It is made available under aCC-BY 4.0 International license.

Fig. S1. Response and differentiation of long-lived naive CD8 T cells after infection.

Experimental design is shown in Figure 1C. (A) The phenotype (KLRG1 and CD127 expression) of progeny derived from fresh and long-lived P14 CD8 T cells in the blood on day 8 post-LCMV-Arm infection. Representative flow cytometry plots were gated on P14 CD8 T cells derived from either fresh- or long-lived-derived population. (B and C) Percentages of progeny derived from fresh and long-live P14 CD8 T cells within total P14 CD8 T cells (B) and their absolute numbers (C) in the spleen at day 98 post-LCMV-Arm infection. Results were representative of 3 or more independent experiments with 4 mice per group. In B and C, each symbol represents an individual mouse, and lines indicate paired comparisons within the same mice. Statistical analysis was performed using paired *t* test. ** $p < 0.01$; *** $p < 0.001$.

bioRxiv preprint doi: https://doi.org/10.1101/2025.10.16.682909; this version posted October 17, 2025. The copyright holder for this preprint (which was not certified by peer review) is the author/funder, who has granted bioRxiv a license to display the preprint in perpetuity. It is made available under aCC-BY 4.0 International license.

Fig. S2. Responses of long-lived naïve CD8 T cells during *Listeria monocytogenes* infection. (A–C) Responses of long-lived P14 CD8 T cells during *Listeria monocytogenes* infection. Long-lived P14 CD8 T cells (1×10^3 cells, $CD45.1^{+/+}$), housed for 40 days in B6 mice, were adoptively co-transferred with an equal number of fresh P14 cells (1×10^3 cells, $CD45.1^+CD45.2^+$) into B6 mice ($CD45.2^{+/+}$), followed by GP33 epitope-expressing *Listeria monocytogenes* infection. Percentages of progeny derived from each population within total P14 CD8 T cells in the blood (A) and Spleen (B). (C) The absolute numbers of memory cells derived from fresh and long-lived P14 cells in the spleen at day 60 post-infection. Data are representative of 2 independent experiments with 4 mice per group. Each symbol represents an individual mouse, and lines indicate paired comparisons within the same mice. Statistical analyses were performed using one-way ANOVA (A) and paired *t* test (B and C). * $p < 0.05$; ** $p < 0.01$; *** $p < 0.001$.

Fig. S3. CD5 and Ly6C expression on fresh and long-lived P14 cells. (A) CD5 and Ly6C expression on long-lived (housed in B6 mice for more than 30 days) and fresh P14 CD8 T cells in the spleen. Representative histograms were gated on $DbGP33^+$ P14 CD8 T cells. (B) Experimental design for panel C. $Ly6C^{Lo}$ and $Ly6C^{Hi}$ long-lived P14 CD8 T cells were sorted, and equal numbers (1×10^3 cells each) of congenically distinct P14 CD8 T cells, including each sorted subset and freshly isolated P14 CD8 T cells, were adoptively co-transferred into B6 mice, followed by LCMV-Arm infection. The responses of sorted and control P14 populations in the blood were analyzed on day 8 post-infection. (C) Normalized frequency of sorted P14 CD8 T cells post-infection. The frequency of control P14 cells at day 8 post-infection was set as 100. Each symbol

bioRxiv preprint doi: https://doi.org/10.1101/2025.10.16.682909; this version posted October 17, 2025. The copyright holder for this preprint (which was not certified by peer review) is the author/funder, who has granted bioRxiv a license to display the preprint in perpetuity. It is made available under aCC-BY 4.0 International license.

represents an individual mouse, bars indicate the mean, and error bars indicate SEM. Statistical analysis was performed using unpaired *t* test. ns, not significant.

Fig. S4. Heterogeneity of naïve CD8 T cells in different tissues. IL-18Rα, CXCR3, and CD73 expression on long-lived (housed in B6 mice for 39 days) and fresh P14 CD8 T cells in the spleen, liver, lymph nodes (LNs), and blood. Representative histograms were gated on DbGP33$^+$ P14 CD8 T cells. inLN, inguinal LN; brLN, brachial LN; meLN, mesenteric LN.

Fig. S5. Cell sorting strategy for Figure 5C. IL-18Rα^{Hi}CD73^{Hi}CD44^{Lo} and IL-18Rα^{Lo}CD73^{Lo}CD44^{Lo} CD8 T cells were sorted from splenocytes of naïve B6 mice.

Fig. S6. Efficiency of Ly6C2 KO in long-lived P14 CD8 T cells. Experimental design is shown in Figure 6D. Ly6C2 KO long-lived P14 CD8 T cells were generated using CRISPR-Cas9. Ly6C expression on WT and Ly6C2 KO long-lived P14 CD8 T cells in the blood on day 8 post-LCMV-Arm infection. Representative histograms were gated on effector P14 CD8 T cells derived from either WT or Ly6C2 KO long-lived P14 CD8 T cells.

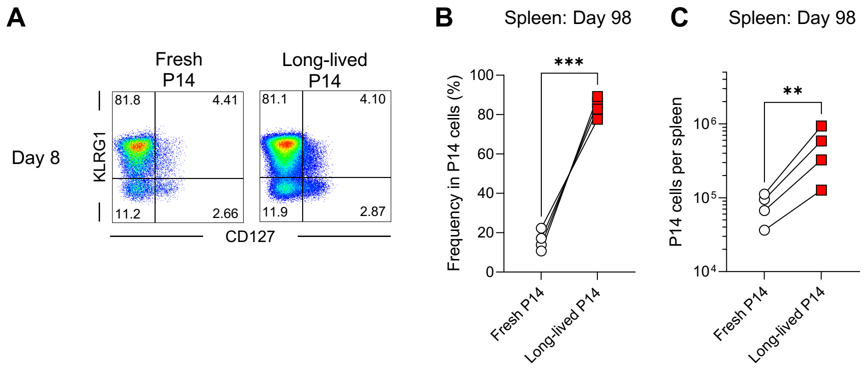
A
Fresh P14
Long-lived P14
Day 8
KLRG1
CD127
81.8
4.41
11.2
2.66
81.1
4.10
11.9
2.87
B
Spleen: Day 98
***
Frequency in P14 cells (%)
100
80
60
40
20
0
Fresh P14
Long-lived P14
C
Spleen: Day 98
**
P14 cells per spleen
10^6
10^5
10^4
Fresh P14
Long-lived P14

Figure S1

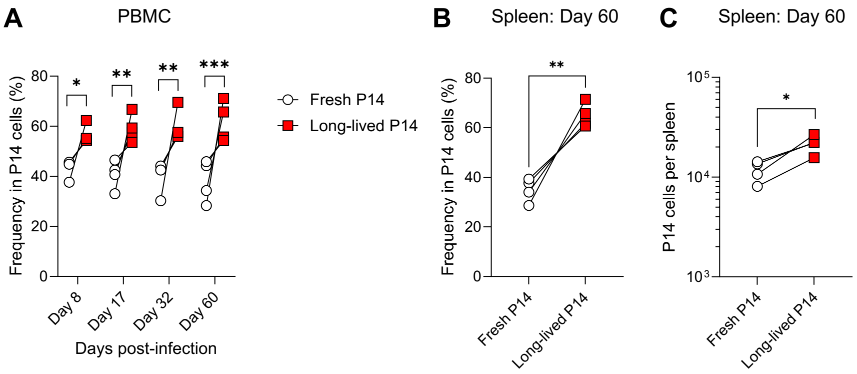
A
PBMC
Frequency in P14 cells (%)
Day 8
Day 17
Day 32
Day 60
Days post-infection
Fresh P14
Long-lived P14
B
Spleen: Day 60
Frequency in P14 cells (%)
Fresh P14
Long-lived P14
C
Spleen: Day 60
P14 cells per spleen
Fresh P14
Long-lived P14

Figure S2

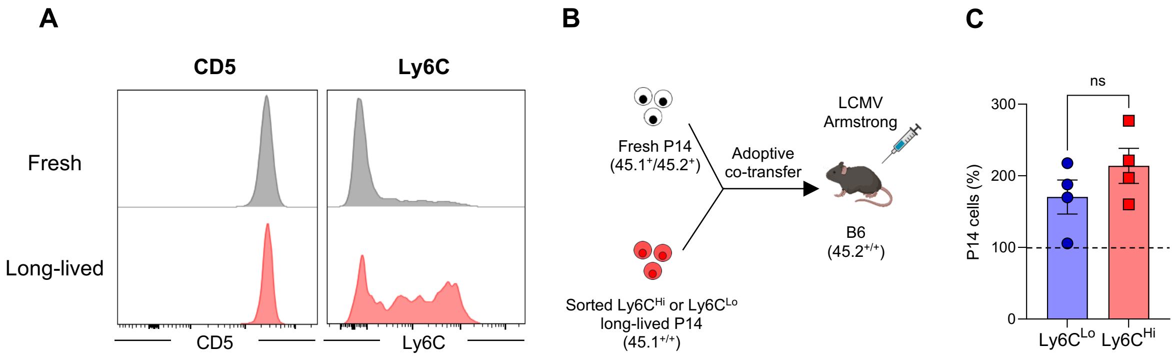
A
CD5
Ly6C
Fresh
Long-lived
CD5
Ly6C
B
Fresh P14
(45.1+/45.2+)
Sorted Ly6CHi or Ly6CLo
long-lived P14
(45.1+/+)
Adoptive
co-transfer
LCMV
Armstrong
B6
(45.2+/+)
C
ns
P14 cells (%)
300
200
100
0
Ly6CLo
Ly6CHi

Figure S3

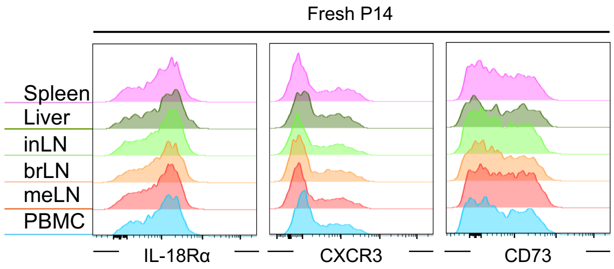
Fresh P14
Long-lived P14
Spleen
Liver
inLN
brLN
meLN
PBMC
IL-18Rα
CXCR3
CD73

Figure S4

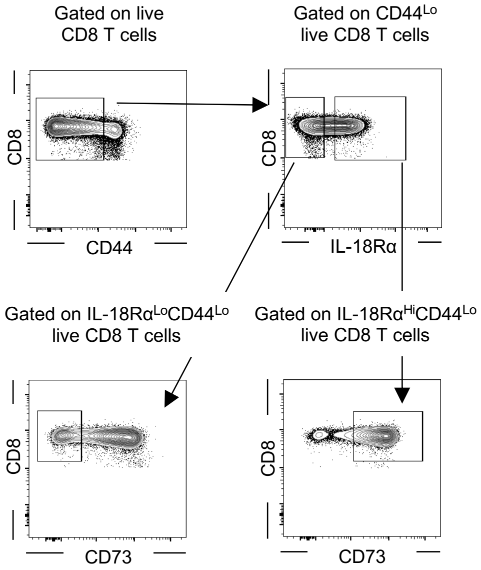

Gated on live
CD8 T cells
Gated on CD44Lo
live CD8 T cells
CD8
CD44
CD8
IL-18Rα
Gated on IL-18RαLoCD44Lo
live CD8 T cells
Gated on IL-18RαHiCD44Lo
live CD8 T cells
CD8
CD73
CD8
CD73

Figure S5

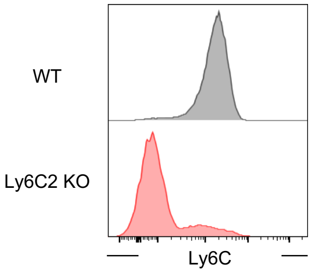

Figure S6
